# Factors associated with food insecurity among pregnant women in Gedeo zone public hospitals, Southern Ethiopia

**DOI:** 10.3389/fpubh.2024.1399185

**Published:** 2024-08-08

**Authors:** Abriham Shiferaw Areba, Denebo Ersulo Akiso, Arega Haile, Belayneh Genoro Abire, Girum Gebremeskel Kanno, Lire Lemma Tirore, Desta Erkalo Abame

**Affiliations:** ^1^School of Public Health, College of Medicine and Health Sciences, Wachemo University, Hossana, Ethiopia; ^2^Department of Statistics, College of Natural Sciences, Dilla University, Dilla, Ethiopia; ^3^School of Public Health, College of Medicine and Health Sciences, Dilla University, Dilla, Ethiopia

**Keywords:** food insecurity, factors, pregnant women, Gedeo zone, public hospitals, Ethiopia

## Abstract

**Background:**

Food insecurity refers to a lack of consistent access to sufficient food for active, better health. Around two billion people worldwide suffer from food insecurity and hidden hunger. This study focuses on food insecurity and associated factors among pregnant women in Gedeo Zone Public Hospitals, Southern Ethiopia.

**Method:**

An institutional-based cross-sectional study was conducted among pregnant women in Gedeo zone public hospitals from May to June 2021. Primary data of 506 pregnant women were collected using interviewer-administered structured questionnaire and a multi-stage sampling technique was used to select study participants. The household food insecurity access scale of the questionnaire was used and a woman was considered as food insecure when it has any of the food insecurity conditions mild, moderate, or severe food insecure, otherwise, it was classified as food secure. Adjusted odds ratio (AOR) and their 95% confidence intervals (CI) determined the association between various factors and outcomes.

**Results:**

Of all study participants, 67.39% of the women were food insecure, and the remaining 32.6% had food security. The pregnant women from rural areas [AOR = 0.532, 95% CI: 0.285, 0.994], married [AOR = 0.232, 95% CI: 0.072, 0.750], had a secondary education [AOR = 0.356, 95%CI: 0.154, 0.822], and be employed [AOR = 0.453, 95% CI: 0.236, 0.872], the wealth index middle [AOR = 0.441, 95% CI: 0.246, 0.793] and rich [AOR = 0.24, 95% CI: 0.128, 0.449] were factors associated with food insecurity.

**Conclusion:**

The study area had a high prevalence of food insecurity. Food insecurity was reduced in those who lived in rural areas, were married, had a secondary education, were employed, and had a wealth index of middle and rich.

## Introduction

Food insecurity refers to a lack of sufficient food, as well as restrictions on the quality, quantity, and/or frequency of food consumption (1–4). Goal 2 of the Sustainable Development Goals (SDGs) aims to end hunger, increase food security, and promote sustainable agriculture. Target 2.1 of the SDGs is aimed at achieving the objective of ending hunger and ensuring year-round access to food for all people, including pregnant and lactating mothers, by 2030 (5).

At the worldwide level, gender differences in the incidence of moderate and severe food insecurity have expanded in the year of the COVID-19 pandemic, with women experiencing 10% more moderate or severe food insecurity in 2020 than males, compared to 6% in 2019. Severe food insecurity affects 28.7% of the population in Eastern Africa, while moderate to severe food insecurity affects 65.3 percent of the population (6).

According to the FAO estimate for 2021, over 2 billion people worldwide are afflicted by moderate food insecurity and hunger, with Sub-Saharan Africa having the highest prevalence (21 percent of the population) (6, 7). According to recent studies in Ethiopia, 7% of households experience severe food insecurity, while 11 and 22% of households experience mild and moderate food insecurity, respectively (8). According to various studies, Food insecurity has been linked to poor pregnancy outcomes, including low birth weight, gestational diabetes, and pregnancy problems (9–16). A study conducted in the United States showed that maternal food insecurity was associated with an increased risk of certain birth defects, such as cleft palate, transposition of the great arteries, and Anencephaly (17).

Furthermore, young children from food-insecure families have poorer general health (18–20), are more likely to be hospitalized (19, 21), have lower levels of parent–child attachment, and experience developmental delays (22–24). Food insecurity and food shortages are associated with poor general, mental, and physical health in women. A study in the USA indicated that food insecurity was associated with women’s reduced mental health. Mental symptoms including depression, stress, and anxiety were associated with household food insecurity in a dose–response relationship and were increased with worsening the food insecurity status (25).

Food insecurity has a substantial effect on the physical health of both the pregnant woman and her child, directly compromising the nutritional state and serum profile of micronutrients, such as iron. It may also trigger a series of stressful events in the family environment due to the difficulty in obtaining food, provoking deterioration in maternal mental health and consequent development of anxiety and depression, and also leading to negative outcomes concerning childcare (8).

Household food insecurity is expected to vary depending on the household head’s gender, age, and level of education; the size of the household; the quantity of livestock held; and financial and human capital-related issues (26). Because food security affects a pregnant woman’s nutritional condition, which is a significant environmental risk factor for poor pregnancy outcomes, securing a sufficient food supply for pregnant women has been a top priority for concerned parties.

However, the risk of food insecurity in pregnant women in this particular study area does not identify at the household level/pregnant women. This causes difficulty in identifying those women who require targeted intervention, aid, and risk of food insecurity at women to work. Though there is a continually high magnitude of food insecurity in Ethiopia, published research does not give significant evidence on its risk factors in all parts of the country. Most surveys done in Ethiopia had a lesser number of research participants and were not conducted on a large scale, making them ineffective for identifying risk factors. This problem motivates the authors to conduct a study supported by an appropriate statistical model on this current crucial issue. Though there is a continually high magnitude of food insecurity in Ethiopia, published research does not give significant evidence on its risk factors in all parts of the country. Most surveys done in Ethiopia had a lesser number of research participants and were not conducted on a large scale, making them ineffective for identifying risk factors. Therefore, this study aimed to assess the magnitude of food insecurity and its associated factors among pregnant mothers in Gedeo zone public Hospitals, in Southern Ethiopia.

## Methods

### Study design and area description

An institution-based cross-sectional study was conducted in Gedeo Zone Public Hospitals in Southern Ethiopia. Gedeo zone is located 369 km from Addis Ababa to the south on the Addis Ababa-Moyale international road and 90 km from Hawassa (Capital city of the region) in the south Nation Nationality and People regional state. The Zone has 1 referral hospital, 3 primary hospitals (Bule, Gedeb, and Yerga Chefe), 38 health centers, 146 health posts, 4 NGO clinics, and 17 reported private health facilities.

### Source population and study population

All pregnant mothers attending antenatal care in Gedeo Zone Health facilities were the source population, while pregnant mothers attending antenatal care in Gedeo Zone Public Hospitals were the study population.

### Determination of sampling size and sampling procedure

The sample size used for this investigation was estimated and computed using a single population proportions method with the following assumptions: 32.4% FI among nursing moms, 95 percent confidence level of 1.96, margin of error of 0.05, and design effect of 1.5. As a result, the study’s ultimate sample size was 506 participants (27). The study subjects were selected using a multi-stage sampling technique. Proportional allocation of the sample was done to each Hospital based on the number of pregnant women available in the Hospital. After consent, one mother was randomly selected from among the pregnant women who matched the eligibility criteria to participate in the study.

### Data collection and procedures

The study data collection instruments were developed after searching PubMed, Google Scholar, Hinari, and the Lancet series for various types of literature. The data was collected using a standardized interviewer-administered questionnaire. The questionnaire was written in English, then translated into Amharic, and then returned to English by language experts to ensure consistency and correctness. Six diploma nurses who were proficient in the local language (Gedeo’ufa) as data collectors and two BSc midwives as supervisors were hired based on their past data-collecting experience.

Nine standard Household Food Insecurity Access Scale (HFIAS) questions derived from the Food and Nutrition Technical Assistance (FANTA) project were used to determine the outcome variable, food insecurity. The instrument consists of nine questions that illustrate the frequency of occurrence and quantify the severity of food insecurity in the previous 4 weeks using Likert scale responses [0 = Never, 1 = rarely (1 or 2 times), 2 = occasionally (3–10 times), and 3 = frequently (>10 times)]. The pregnant ladies were required to respond to these questions on behalf of their entire household. At the time of data collection, this technique was used to assess food access for all family members. The nine items ranging from 0 to 27 were used to compute the cumulative score of food insecurity among expectant mothers, with a higher score indicating that the household members experienced more food insecurity. All “Yes” responses were coded as 1 and “No” responses were coded as 0, and the responses were totaled to determine the level of household food insecurity (28).

The household’s wealth index was calculated using Principal Component Analysis (PCA) and took into account the latrine, water source, household assets, livestock, and ownership of agricultural land. All non-dummy variables’ responses were divided into three groups. The highest score was given a 1 rating. The two lower values, on the other hand, were given code 0. The variables with a commonality value larger than 0.5 were used to generate factor scores in PCA. Finally, the wealth score was calculated using each household’s score on the first major component. The wealth score was divided into three quintiles to classify households as low, medium, or wealthy.

### The study’s variables

The following are the response and predictor variables considered in the model for parameter estimation.

#### Response variable

Food insecurity among pregnant women is the study’s outcome variable. If the women are food insecure, this can be dichotomized as 1 and 0 correspondingly.

#### Explanatory variables

The Table 1 lists the predictor variables that were investigated in this study to investigate food insecurity among pregnant women.

**Table 1:** Description of independent variables used in the study.

Covariates	Description	Categories
Age	Age of pregnant women	0 = 15–19, 1 = 20–24, 2 = 25–29, 3= ≥ 30
Woreda	Woreda of pregnant women	0 = Dilla, 1 = Gedebe, 2 = Yirga Chafe, 3 = Bule, 4 = Others
Residence	Residence of women who live	0 = Urban 1 = Rural
Education	Educational level of women	0 = No education, 1 = Primary, 2 = Secondary, 3 = Higher
Marital status	Marital status of women	0 = Others (Single, Divorce), 1 = Married
Employment	Employment status of women	0 = Unemployed, 1 = Employed
Wealth index	Wealth Index of Women	1 = Poor, 2 = Middle, 3 = Rich
Family size	No. of a family member	1 = 2, 2 = 3–4, 3 = 5 and above
Parity	No. of live birth	0 = No Birth, 1 = One Birth, 2 = Two Birth, 3 = Three and above birth
Gravid	No. of pregnancy	1 = First pregnancy, 2 = 2–3 Pregnancy, 3 = 4 and above
Willingness	Pregnancy willingness	0 = Unwanted, 1 = Wanted
Complication	Pregnancy Complication	0 = No, 1 = Yes
Food extension	Food Extension Service	0 = No, 1 = Yes

### Eligibility criteria

#### Inclusion criteria

All Pregnant mothers of pregnancy attending ANC services at selected health institutions were included in this study.

#### Exclusion criteria

Pregnant mothers’ co-morbidities with complications and special requirements were excluded from the study. Diagnosed with chronic diseases like diabetes, hypertension, and twin pregnancy.

### Operational definitions

#### Food secure women

Women who have experienced none of the Food Insecurity (access) conditions or have just been worried, although rarely, during the past 4 weeks (28).

#### Food insecure women

Women who are unable at all times to access food sufficient to lead an active and healthy life (includes all stages of FI; mild, moderate, and severe) (28).

#### Mildly food insecure women

Women who worry about not having enough food sometimes or often and/or are unable to eat preferred foods and/or eat a more monotonous diet than desired and/or some foods considered undesirable, but only rarely (28).

#### Moderately food insecure women

Women who sacrifice quality more frequently, by eating a monotonous diet or undesirable foods sometimes or often, and/or have started to cut back on quantity by reducing the size of meals or number of meals, rarely or sometimes. However, they do not experience any of the three most severe conditions (28).

#### Severely food insecure women

Women who have been forced to cut back on the meal size or number of meals often and experience any of the three most severe conditions (running out of food, going to bed hungry, or going a whole day and night without eating) (28). A woman was considered as food insecure when it has any of the food insecurity conditions mild, moderate, or severe food insecure, otherwise, it was classified as food secure (28).

#### Wealth status

A reliability test was performed using the economic variables involved in measuring wealth. The variables that were employed to compute the principal component analysis, at the end of the principal component analysis, the wealth index was obtained as a continuous scale of relative wealth. Finally, the Percentile group of the wealth index was created to group under three wealth categories, poor, middle, and rich (29).

### Data quality control

Before data collection, the questionnaire was first written in English, then translated into Amharic, and then back to English for consistency. The purpose, methodology of the research on food insecurity, data collecting and interviewing style, and data recording were all covered in two days of training one week previous to the day of data collection. In a health center outside of the study area, the questionnaire was pre-tested on 5% of actual respondents. The supervisors and primary investigators kept a close eye on the overall activities during the data collection period to guarantee that the data was of high quality. Before analysis, all of the obtained data were double-checked, coded, entered into SPSS version 25, and cleaned to eliminate inconsistencies and incompleteness. The STATA/SE statistical software package version 14.0 was used to analyze the data.

### Data analysis

Descriptive statistics was used to report the distribution of the data among variables using frequency and percentage. A bi-variable logistic regression analysis was performed to assess associations between each independent variable and food insecurity. A multivariable model should include all covariates relevant in bi-variable analyses at the *p* = 0.20 to 0.25 level from the start. In a multivariable model, the variables that tend to be relevant from the bi-variable analysis are fitted together. For multivariate analysis, statistical significance was determined using a 95% confidence interval and a *p*-value of less than 0.05. As a consequence, backward exclusion is used to omit non-significant variables from the final model (30).

## Results

A total of 506 pregnant moms were considered in this investigation. Food insecurity and food security were found in 67.4% and 32.6 percent of those moms, respectively. There were 139 (27.47 percent) and 367 (72.53 percent) women from rural and urban areas, respectively, with rural residents experiencing 108 (21.34 percent) less food insecurity than urban residents experiencing 233 (46.05 percent).

When it comes to the age of the mothers, the minimum number of women discovered in the age group of 15–19 years is 26 (5.14%), while the greatest number of women found in the age group of 20–24 years is 224 (44.27 percent). 478 (94.47 percent) of the moms in the research were married, whereas 28 (5.53) were unmarried (single and divorced) (Table 2).

**Table 2:** Descriptive summaries of food insecurity among pregnant mothers in Gedeo zone public hospitals.

Covariates	Categories	Food insecurity status		No. out of 506 (%)
		Food secure (%)	Food insecure (%)	
Residence	Rural	31 (6.13)	108 (21.34)	139 (27.47)
	Urban	134 (26.48)	233 (46.05)	367 (72.53)
Age (year)	15–19	8 (1.58)	18 (3.56)	26 (5.14)
	20–24	75 (14.82)	149 (29.45)	224 (44.27)
	25–29	61 (12.06)	106 (20.95)	167 (33)
	30 and above	21 (4.15)	68 (13.44)	89 (17.59)
Marital status	Unmarried	4 (0.79)	24 (4.74)	28 (5.53)
	Married	161 (31.82)	317 (62.65)	478 (94.47)
Educational status	No Education	13 (2.57)	82 (16.21)	95 (18.77)
	Primary	54 (10.67)	141 (27.87)	195 (38.54)
	Secondary	55 (10.87)	77 (15.22)	132 (26.09)
	Higher	43 (8.50)	41 (8.10)	84 (16.60)
Employment status	Unemployed	112 (22.13)	303 (59.88)	415 (82.02)
	Employed	53 (10.47)	38 (7.51)	91 (17.98)
Wealth index	Poor	24 (4.74)	144 (28.46)	168 (33.20)
	Middle	58 (11.46)	111 (21.94)	169 (33.40)
	Rich	83 (16.40)	86 (17)	169 (33.40)
Family size	2	37 (7.31)	86 (17)	123 (24.31)
	3–4	83 (16.40)	146 (28.85)	229 (45.26)
	5 and above	45 (8.89)	109 (21.54)	154 (30.43)
Parity	No Previous Birth	50 (9.88)	112 (22.13)	162 (32.02)
	1 Previous Birth	54 (10.67)	86 (17)	140 (27.67)
	2 Previous Birth	29 (5.73)	59 (11.66)	88 (17.39)
	3 and above	32 (6.32)	84 (16.60)	116 (22.92)
Gravid	1 Pregnancy	45 (8.89)	110 (21.74)	155 (30.63)
	2–3 Pregnancies	87 (17.19)	147 (29.05)	234 (46.25)
	4 and above	33 (6.52)	84 (16.60)	117 (23.12)
Pregnancy willingness	Unwanted	10 (1.98)	25 (4.94)	35 (6.92)
	Wanted	155 (30.63)	316 (62.45)	471 (93.08)
Complication of pregnancy	No	152 (30.04)	304 (60.08)	456 (90.12)
	Yes	13 (2.57)	37 (7.31)	50 (9.88)
Food extension service	No	142 (28.06)	284 (56.13)	426 (84.19)
	Yes	23 (4.55)	57 (11.26)	80 (15.81)

Unmarried (single, divorced).

A single covariate binary logistic regression model analysis is an appropriate approach for screening out potentially essential variables before including them directly in a multivariable model. Each covariate’s association with food insecurity among pregnant women was discussed. Food insecurity among pregnant mothers was significantly associated with place of residence, marital status, educational status, employment status, wealth index, family size, parity, and gravidity, but age of the mother, pregnancy willingness, pregnancy complications, and food extension service were not significant at a modest level of significance of 0.25 (Table 3).

**Table 3:** Prevalence of food insecurity, based on individual FIAS among pregnant mothers in Gedeo zone public hospitals.

Nine FIAS	Yes	Rarely	Sometimes	Often
	Freq (%)	Freq (%)	Freq (%)	Freq (%)
Worry about food	215 (42.5)	56 (11.1)	125 (24.7)	34 (6.7)
Unable to eat preferred foods	204 (40.3)	64 (12.6)	101 (20)	39 (7.7)
Eat a limited variety of foods	184 (36.4)	65 (12.8)	92 (18.2)	27 (5.3)
Eat foods they really do not want eat	181 (35.8)	52 (10.3)	69 (13.6)	60 (11.9)
Eat a smaller meal	181 (35.8)	57 (11.3)	90 (17.8)	34 (6.7)
Eat fewer meals in a day	198 (39.1)	50 (9.9)	111 (21.9)	37 (7.3)
No food of any kind in the household	194 (38.3)	54 (10.7)	99 (19.6)	41 (8.1)
Go to sleep at night hungry	194 (38.3)	56 (11.1)	101 (20)	37 (7.3)
Go a whole day and night without eating	206 (40.7)	57 (11.3)	74 (14.6)	75 (14.8)

FIAS, Food Insecurity Access Scale; Freq, frequency.

**Figure 1:**
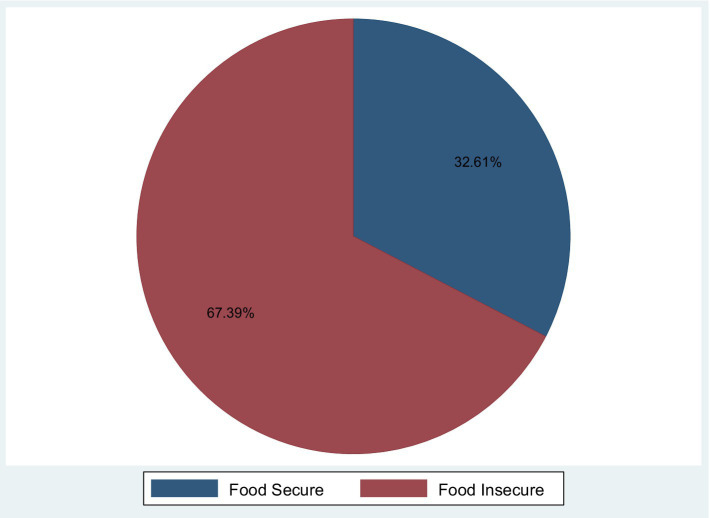
Magnitude of food insecurity among pregnant women in Gedeo zone public hospitals, Southern Ethiopia.

In the Gedeo zone Public Hospitals, the place of residence, marital status, educational status, employment status, average monthly income, and wealth index all had statistically significant effects on food insecurity, i.e., the confidence interval for the adjusted odds ratio did not include one and the *p*-value was less than 0.05. The estimated odds ratio for pregnant mothers from rural areas were 0.532 when other predictor factors were kept constant in a multivariable regression model. This means that pregnant mothers from rural areas were 0.532 times (AOR = 0.532, 95% CI: 0.285, 0.994) less likely than mothers from urban areas to be food insecure (Figure 1).

Food insecurity was reduced by 76.8% in expectant pregnant mothers who were married compared to those who were not (single, divorced) (AOR = 0.232, 95 percent CI: 0.072, 0.750). When the influence of other factors was held constant in the model, pregnant mothers with a secondary education had a 35 percent lower risk of food insecurity than pregnant mothers without a secondary education (AOR = 0.35, 95 CI: 0.154, 0.822). With the effect of other independent variables constant in the model, pregnant mothers who had employment status were 0.453 times (AOR = 0.453, 95% CI: 0.236 to 0.872) less likely to have food insecurity than those who had unemployment status.

When other predictor variables in the regression model were held constant, pregnant women with middle and high economic status were 0.441 and 0.24 times less likely to have food insecurity than those with low economic status (AOR = 0.441, 95 percent CI 0.246 to 0.793) and (AOR = 0.24, 95 percent CI 0.128 to 0.449) respectively (Table 4).

**Table 4:** Multivariable logistic regression analysis for food insecurity among pregnant mothers in Gedeo zone public hospitals.

Covariates	$\hat{\beta }$	SE	Z	Sig	AOR	95% CI for AOR	
Place of residence							
Urban^®^							
Rural	−0.631	0.319	−1.98	0.048	0.532	0.285	0.994
Marital status							
Others (single, divorced)^®^							
Married	−1.463	0.599	−2.44	0.015	0.232	0.072	0.750
Educational status							
No education^®^							
Primary	−0.542	0.378	−1.44	0.151	0.581	0.277	1.22
Secondary	−1.033	0.427	−2.42	0.016	0.356	0.154	0.822
Higher	−0.583	0.509	−1.15	0.252	0.558	0.206	1.513
Employment status							
Unemployed^®^							
Employed	−0.791	0.333	−2.37	0.018	0.453	0.236	0.872
Wealth index							
Poor^®^							
Middle	−0.818	0.298	−2.67	0.006	0.441	0.246	0.793
Rich	−1.429	0.321	−4.45	0.000	0.240	0.128	0.449
Constant	1.62	0.806	1.01	0.000	5.030	3.450	6.610

$\hat{\beta }\text{,}$ regression coefficients; SE, standard error; 95% CI for AOR, 95% confidence interval for adjusted odds ratio; ®, reference.

## Discussion

Food security and proper nutrition are essential for human growth and development, which necessitates access to enough, diverse, and high-quality food resources (25). In terms of food insecurity, 67.39% with (95% CI: 63.3, 71.5%) of pregnant women in this survey were food insecure. The findings of this study were similar to those of studies conducted in Hossana (67.5 percent) (31) and Areka (69.6 percent) (32). On the other hand, it is greater than the Atay District (36.8%) (33), Abay District (38.1%) (8), and Sodo town (37.6%) studies completed in Ethiopia (34).

Seasonal variations in family food security status, which are frequently higher in Ethiopia’s summarizing season, could explain the increased degree of food insecurity. It could also be explained by households having fewer and smaller meals as a result of a monotonous diet and a lack of variety in food items. These discrepancies could be related to variances in the study participants’ socio-demographic variables. Seasonal fluctuation may be another major explanation for the apparent difference, as this study was conducted during the summer season whereas the other experiments were conducted during the pre-harvest season.

According to the findings, rural residency, marriage, secondary education, and wealth index intermediate and rich were all significant predictors of food insecurity among pregnant women. The location of residence was found to be a major differential for food insecurity in this study, and the findings suggest that moms from urban areas are more likely to have food insecurity than mothers from rural areas. Previous research backs up this conclusion (35).

Pregnant mothers who were part of a married pair were less likely to be food insecure than those who were single or divorced. This could be because married households in the study area had better access to farmland and social security than unmarried households. This was supported by research (34, 36). Mothers’ educational status is one of the determinants of their food insecurity.

This implies that women with secondary education were less likely to be food insecure than those without. Previous research backs up this research (25, 33, 36–38). This is because educated mothers are more likely to know how to create, improve, manage, and produce enough varieties of farms to ensure their families and their own food security. Employment status is one factor that influences food insecurity among pregnant women. The findings reveal that expectant moms who are employed are less likely to be food insecure than those who are unemployed. The findings of this study were similar to those of others (25, 31, 38, 39).

Pregnant women with medium and upper-class economic positions were less likely to experience food insecurity than mothers with lower-class economic status. i.e., poor pregnant women were more likely to be food insecure as a result of their low wealth index. This could be explained by the fact that poor pregnant women may have no or only one source of income, making it difficult for them to purchase appropriate foods to meet the demands of their household members owing to extreme poverty. This finding was in line with earlier research (31–33, 40).

### Limitations of the study

Some variables that can affect the food insecurity of women; are knowledge level, dietary practice, and perception of the participants which were not addressed in this study. Since the study depends on self-reporting, there might be social desirability and recall bias from respondents. In addition, the predictors of food insecurity may not necessarily have a cause-and-effect relationship with food insecurity because the study design was cross-sectional. Further research with strong study designs will also need to come through seasonal variations of household food insecurity among pregnant women and also use advanced statistical models like multilevel models using individual level and community level variables to identify variation of each level.

## Conclusion

The present study revealed a high level of food insecurity among pregnant mothers’ households. Place of residence, marital status, educational status, employment status, and wealth index were factors significantly associated with food insecurity. Rural residence, marriage, secondary education level, and wealth index intermediate and rich were reduced significant predictors of food insecurity.

## Data availability statement

The raw data supporting the conclusions of this article will be made available by the authors, without undue reservation.

## Ethics statement

The studies involving humans were approved by the Dilla University Institutional Research and Ethical Review Board. The studies were conducted in accordance with the local legislation and institutional requirements. The participants provided their written informed consent to participate in this study.

## Author contributions

AA: Conceptualization, Data curation, Formal analysis, Funding acquisition, Investigation, Methodology, Project administration, Resources, Software, Supervision, Validation, Visualization, Writing – original draft, Writing – review & editing. DenA: Funding acquisition, Resources, Supervision, Validation, Visualization, Writing – original draft, Writing – review & editing. AH: Writing – review & editing, Conceptualization, Data curation, Investigation, Methodology, Resources, Visualization, Writing – original draft. BA: Funding acquisition, Resources, Validation, Visualization, Writing – original draft, Writing – review & editing. GK: Conceptualization, Formal analysis, Funding acquisition, Investigation, Project administration, Resources, Validation, Visualization, Writing – review & editing. LT: Formal analysis, Funding acquisition, Project administration, Resources, Validation, Visualization, Writing – original draft, Writing – review & editing. DesA: Data curation, Formal analysis, Funding acquisition, Methodology, Project administration, Resources, Supervision, Validation, Visualization, Writing – original draft, Writing – review & editing.
